# Public health round-up

**DOI:** 10.2471/BLT.20.010620

**Published:** 2020-06-01

**The need to maintain essential services**

A mother shows the anti-retroviral medication that she takes daily for human immunodeficiency virus (HIV) infection. According to a modelling exercise published 11 May, a six-month disruption of antiretroviral therapy could see an increase of 500,000 HIV-related deaths in the African Region.

**Figure:**
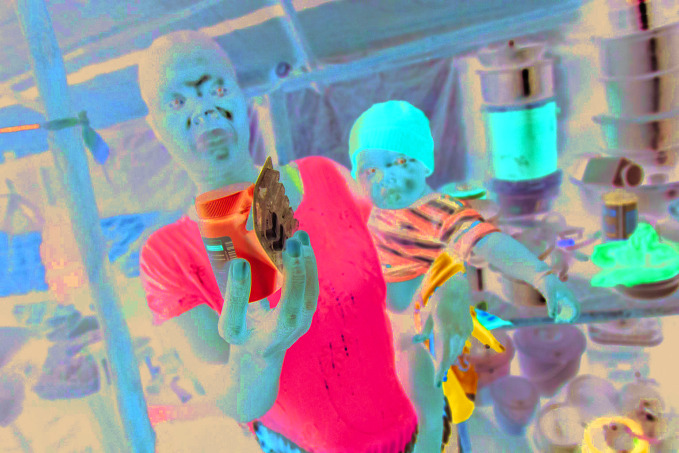
UNICEF/ Albert Gonzalez Farran

## Accelerating COVID-19 products

Heads of state and global health leaders came together virtually on 24 April to launch the Access to COVID-19 Tools Accelerator, a global collaboration to accelerate the development and production of vaccines, tests and treatments for COVID-19 and to ensure assure equitable access to those products worldwide.

The virtual event was co-hosted by the World Health Organization (WHO), the President of France, the President of the European Commission, and the Bill & Melinda Gates Foundation, and was joined by the United Nations Secretary General, the African Union Commission Chairperson, the Group 20 President and heads of state.

Representatives from key stakeholders, including the Global Fund, Unitaid, the Wellcome Trust, and the Developing Countries Vaccine Manufacturers’ Network, committed to supporting the initiative.

Since January, WHO has been working with researchers from hundreds of institutions to develop and test vaccines, standardize assays and regulatory approaches on innovative trial designs and define criteria to prioritize vaccine candidates.

“Our shared commitment is to ensure all people have access to all the tools to prevent, detect, treat and defeat COVID-19,” said Tedros Adhanom Ghebreyesus, WHO Director-General. “No country and no organization can do this alone.”

https://bit.ly/35yFoGC

## Partnership for pandemic and beyond

WHO and the European Investment Bank established a partnership to respond to the COVID-19 pandemic and other health challenges.

Announced on 1 May, the partnership will focus on financing immediate COVID-19-related needs, including the supply of personal protective equipment, diagnostics and clinical management capacity. Longer term, the partnership will target investment to health preparedness and primary health care, with a focus on health workforce, infrastructure, and water, sanitation and hygiene.

In the first phase of the collaboration, the partnership will address urgent needs and strengthen primary health care in ten African countries.

The European Investment Bank is the long-term lending institution of the European Union and is owned by the European Union Member States.

https://bit.ly/2W9o1sA

## Ebola persisting

The Democratic Republic of the Congo’s Ebola epidemic continues to take lives. As of 7 May, seven people had been confirmed to be infected with the Ebola virus since the outbreak’s 10 April resurgence. The infections were reported in the Kasanga, Malepe and Kanzulinzuli health areas in the Beni health zone. Four of the people confirmed to have Ebola died, including two who died in the community. As of 7 May, the origin of this cluster of cases had not been confirmed.

The outbreak was declared in August 2018. As of 5 May 2020, a total of 3462 people had been infected with the virus, 2279 of whom had died.

https://bit.ly/3bnGRAJ

## Cost of HIV inaction

The disruption of antiretroviral therapy for human immunodeficiency virus (HIV) during the COVID-19 pandemic could set back the clock on HIV-related deaths in sub-Saharan Africa to 2008, when more than 950 000 HIV-related deaths were reported in the region.

The prediction was made by a modelling group convened by WHO and the Joint United Nations Programme on HIV and AIDS (UNAIDS), which released its findings on 11 May. The group estimated that if efforts are not made to mitigate interruptions in health services and supplies during the COVID-19 pandemic, a six-month disruption of antiretroviral therapy could lead to more than 500 000 extra deaths from HIV-related illnesses, including from tuberculosis, in sub-Saharan Africa in 2020–2021.

Some countries are already taking steps to address the problem, for example by allowing people to collect bulk packs of treatment, and other essential commodities, including self-testing kits, from drop-off points, which relieves pressure on health services and the health workforce.

https://bit.ly/3cqsgFX

## COVID-19 human challenge guidance

WHO released ethics guidance on controlled human infection studies (human challenge studies) for COVID-19. Released on 6 May, the guidance presents eight ethical criteria for conducting human challenge studies. The authors recommend that for these studies to proceed, it should be demonstrated that all eight criteria have been satisfied and suggest that initial studies be limited to healthy adults aged between 18 and 30.

Human challenge studies involve the deliberate infection of healthy volunteers and can be particularly valuable for testing vaccines. They can also be substantially faster to conduct than vaccine field trials and can be used to compare the efficacy of multiple vaccine candidates, and facilitate selection of promising candidate vaccines for larger studies.

https://bit.ly/2LjxtUo

## COVID-19 in Africa

Between 29-44 million people could become infected with SARS-CoV-2 in Africa in the first year of the pandemic if containment measures fail. The prediction, published on 7 May in a study by the WHO Regional Office for Africa is based on modelling and considers 47 countries in the WHO African Region.

The study also predicts as many as 190 000 deaths from COVID-19 and recommends that countries strengthen health system capacity, particularly of primary hospitals, and ensure that basic emergency care is included in primary health systems.

“The importance of promoting effective containment measures is ever more crucial, as sustained and widespread transmission of the virus could severely overwhelm our health systems,” said Dr Matshidiso Moeti, the WHO Regional Director for Africa.

https://bit.ly/2Wndquq

## Tackling COVID-19 in prisons

WHO released a checklist designed to support Member States in effective preparedness, prevention and control of COVID-19 in prisons and other places of detention. Released on 7 May, the checklist covers actions specifically designed or adapted to tackle the current COVID-19 pandemic, as well as elements relating to wider service planning and delivery.

The checklist includes: risk assessment and risk management – to reduce the chances of COVID-19 spreading in prisons; effective referral systems, clinical management, and training of prison staff.

https://bit.ly/3dujp6c

## Emergency Financing for COVID-19

The Pandemic Emergency Financing Facility allocated US$ 195.84 million to 64 of the world’s poorest countries. Announced on 27 April, the funds will be directed to areas with the most vulnerable populations, especially in fragile and conflict-affected countries and will go towards purchasing essential and critical lifesaving medical equipment, personal protective equipment, therapeutics and medicine, and paying health workers on the frontlines of the crisis.

The facility is a World Bank fund designed to provide financing to help the world’s poorest countries respond to cross-border, large-scale outbreaks.

https://bit.ly/3fnnAmd

**Cover Photo**

A boy listens to a radio-broadcast class in the village of Morovine, in the North of Côte d'Ivoire. Since the COVID-19 pandemic began UNICEF has been working with the Ministry of Education in Côte d'Ivoire, on a home schooling initiative that includes taping lessons to be aired on national television and radio.

**Figure:**
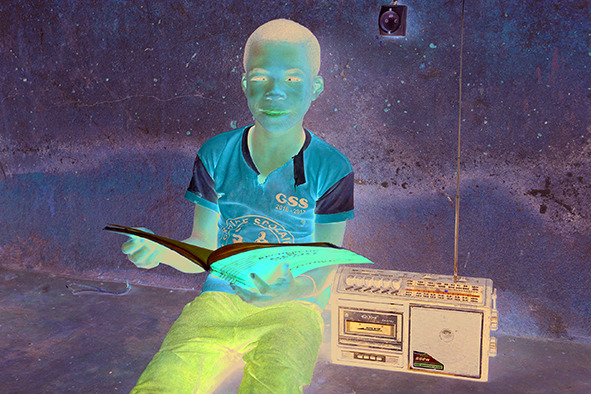
UNICEF / Frank Dejongh

## World health statistics

WHO launched the *World health statistics 2020 report* on 13 May. Compiling statistics for 194 Member States, the report summarizes trends in life expectancy and causes of death and reports data for 46 health and health-related sustainable development goal targets.

The 2020 report reveals that life expectancy and healthy life expectancy both increased by over 8% globally between 2000 and 2016. However, both indicators remain influenced by income, while also reflecting progress Member States are making towards universal health coverage.

The report confirms the widely observed shift in the disease burden from infectious to noncommunicable diseases (NCDs), particularly in low- and middle-income countries where delivery of effective NCD interventions remains a major challenge to health systems.

https://bit.ly/2LsMgvG

## Smallpox anniversary

The 40th anniversary of smallpox eradication was celebrated on 8 May. The World Health Assembly officially declared smallpox to be eradicated on 8 May 1980, marking the end of a disease that had killed 300 million people in the 20th century alone.

Smallpox eradication was achieved thanks to a 10-year global effort that was led by WHO and involved thousands of health workers administering half a billion vaccinations.

Speaking at a virtual event hosted at WHO headquarters, WHO Director-General, Tedros Adhanom Ghebreyesus said, “As the world confronts the COVID-19 pandemic, humanity’s victory over smallpox is a reminder of what is possible when nations come together to fight a common health threat.”

https://bit.ly/2xOqOhA

**Looking ahead**

14 June - World Blood Donor Day. Virtual event due to COVID-19. Global host, Sri Lanka

6 – 10 July - International AIDS Conference. Virtual event due to COVID-19.

